# Granulomatosis With Polyangiitis (GPA) Mimicking Metastatic Malignancy

**DOI:** 10.7759/cureus.71009

**Published:** 2024-10-07

**Authors:** Abraham Mohan, Supriya M Kuriakose, Lijo James, Bindu T G., Suman Jangra, Alisha T Jose, Deepu M

**Affiliations:** 1 Rheumatology, Caritas Hospital and Institute of Health Sciences, Kottayam, IND; 2 Otorhinolaryngology and Audiology, Caritas Hospital and Institute of Health Sciences, Kottayam, IND; 3 Neurology, Caritas Hospital and Institute of Health Sciences, Kottayam, IND; 4 Laboratory, Caritas Hospital and Institute of Health Sciences, Kottayam, IND; 5 Nuclear Medicine, DDNMRC PET Scans Pvt. Ltd., Kottayam, IND; 6 Pulmonology, Caritas Hospital and Institute of Health Sciences, Kottayam, IND

**Keywords:** acute suppurative otitis media, c-anca and pr3 anca antibodies, metastatic malignancy, necrotizing granulomatous vasculitis, pansinusitis

## Abstract

Granulomatosis with polyangiitis is a rare vasculitis characterized by necrotizing granulomatous inflammation affecting small blood vessels. It often presents with pulmonary masses and nodules, mimicking malignancy in imaging studies. Distinguishing granulomatosis with polyangiitis from cancer is crucial due to differing treatment approaches and prognoses. We describe a 54-year-old male with granulomatosis with polyangiitis presenting with headache, bilateral ear discharge, cough, and weight loss. Imaging revealed metabolically active lesions in the lungs and nasopharynx, initially suggestive of metastatic malignancy. Biopsies from the lung and nasopharyngeal lesions showed necrotizing granulomatous vasculitis, which was suggestive of granulomatosis with polyangiitis. Serological testing was positive for cytoplasmic antineutrophil cytoplasmic antibodies (c-ANCA) with elevated proteinase 3 (PR3) antibodies. This case underscores the diagnostic challenges of granulomatosis with polyangiitis, which can mimic malignancy radiologically. Early recognition and treatment initiation are pivotal in improving patient outcomes and preventing organ damage in granulomatosis with polyangiitis.

## Introduction

Granulomatosis with polyangiitis (GPA) is a small-vessel vasculitis associated with ANCA that can be catastrophic. It is characterized by the involvement of the organs such as the kidney, lungs, and upper respiratory tract [1]. Granulomatous inflammation and necrotizing vasculitis of small blood vessels leads to diverse clinical presentations with a classic triad of symptoms involving the upper and lower respiratory tract as well as the kidneys [1-3]. In 25-80% of cases, pulmonary involvement in GPA is present [4]. The most common radiographic presentation of GPA is characterized by pulmonary masses and nodules, which are often multiple and cavitating [5]. Because of its complex clinical presentation, GPA can be challenging to diagnose and distinguish from other medical conditions like sarcoidosis, infection, or malignancy [5]. PET/CT scanning in GPA may be useful in identifying pulmonary involvement and guiding decisions regarding biopsy sites but it cannot distinguish between inflammatory and malignant lesions [6]. The radiographic lesions of GPA may mimic malignancy with spiculation and invasion of the surrounding structures [7]. This case intends to highlight that early tissue biopsy and histopathological examination of mass lesions when present may help in prompt diagnosis and treatment of GPA.

## Case presentation

This case involves a 54-year-old male who presented with a history of headache, bilateral ear pain, and discharge, which lasted for one week at a UAE hospital at the beginning of May 2023. The patient had accompanying weight loss. The patient had no history of fever, epistaxis, or hemoptysis. The patient has had a history of type 2 diabetes mellitus for the past five years. He underwent splenectomy in 2009 following a penetrating abdominal trauma. He quit smoking two years before the onset of this illness. He was evaluated and diagnosed to have bilateral acute suppurative otitis media (ASOM) with acute on chronic sinusitis and was started on oral antibiotics (amoxicillin-clavulanic acid) on 9^th^ May 2023. This treatment was given for one week. He didn’t improve, so he was admitted and treated with intravenous antibiotics (Piperacillin-tazobactam) for the next week. He had persistent headaches and ear discharge despite treatment; hence, he was advised to take a non-contrast CT scan of the paranasal sinuses, which was done on 6^th^ June 2023. It showed bilateral maxillary, ethmoidal, sphenoid, and frontal sinusitis with occlusion of the left osteo-meatal unit. Subsequently, he developed a non-productive cough, for which a chest X-ray was taken on 15^th^ July 2023 and showed a smooth, homogenous opacity in the right upper zone with clear cutoff margins. It was considered to be a right upper lobe segmental consolidation patch. Haziness was seen extending from above the patch to the horizontal fissure, with mild thickening of the horizontal fissure.

A CT chest with contrast was done on the same day, which showed a large heterogeneously enhancing soft tissue/lung parenchymal mass at the right upper lobe extending from the hilum to the apex. It measured 7×5.4 cm in the coronal plane with central hypoattenuating/early necrotic regions. Tiny pulmonary vessels were seen traversing along the mass predominantly in the peripheral regions. The mass had mild spiculated margins and subtle haziness at the adjacent parenchyma. There were some regions of loss of plane between this mass, the adjacent intercostal muscles, and the pleura at the superolateral right apical region. The apical segmental bronchus of the right upper lobe was not well visualized and was probably occluded. At least two other discrete nodules measuring 9 to 11 mm with subtle irregular margins were noted in the anterior right upper lobe and right middle lobe. Pleural-based masses/nodules measuring up to 3.5 cm in the longest axis were noted in the posterior basal segment of the right lower lobe. There was a 12 mm nodule in the medial basal segment of the left lower lobe and a 3.5 mm nodule in the lingular segment of the left upper lobe. Few enhancing closely placed right hilar, pre and subcarinal lymph nodes were noted without discrete fatty hilum. The short-axis diameter of these nodes measured up to 12 mm. Calcification was also seen with these lymph nodes. A few small-volume hilar lymph nodes were also present. His laboratory investigations were done around the same time. Abnormal results included a low hemoglobin of 9.5 g/dl, a raised WBC of 15.5 x10^9^/L, a raised platelet count of 763 x10^9^/L, and an elevated CRP of 14 mg/dl. His renal function tests and liver function tests were normal. The urine protein-creatinine ratio was raised (0.9), but there were no urinary sediments. HBA1C was 8.79%, D-dimer was elevated (982 mg/dl), and ANA (IFA) was done, which was negative. A CT scan of the brain (non-contrast) was repeated on 19^th^ July 2023, which showed features of pansinusitis similar to the previous scan. Bilateral otomastoiditis was present, more on the left side. Lobulated soft tissue was present in the left pharyngeal recess in the fossa of Rosenmuller, causing bulging of Torus tubarius and superiorly extending up to the base of the skull. It measured approximately 2.4 cm×2.4 cm. He was advised to undergo MRI imaging for further characterization of the mass. He thereafter moved from the UAE to our hospital in Kerala, India, as he was told he may be suffering from malignancy and wanted to get evaluated here.

**Figure 1:**
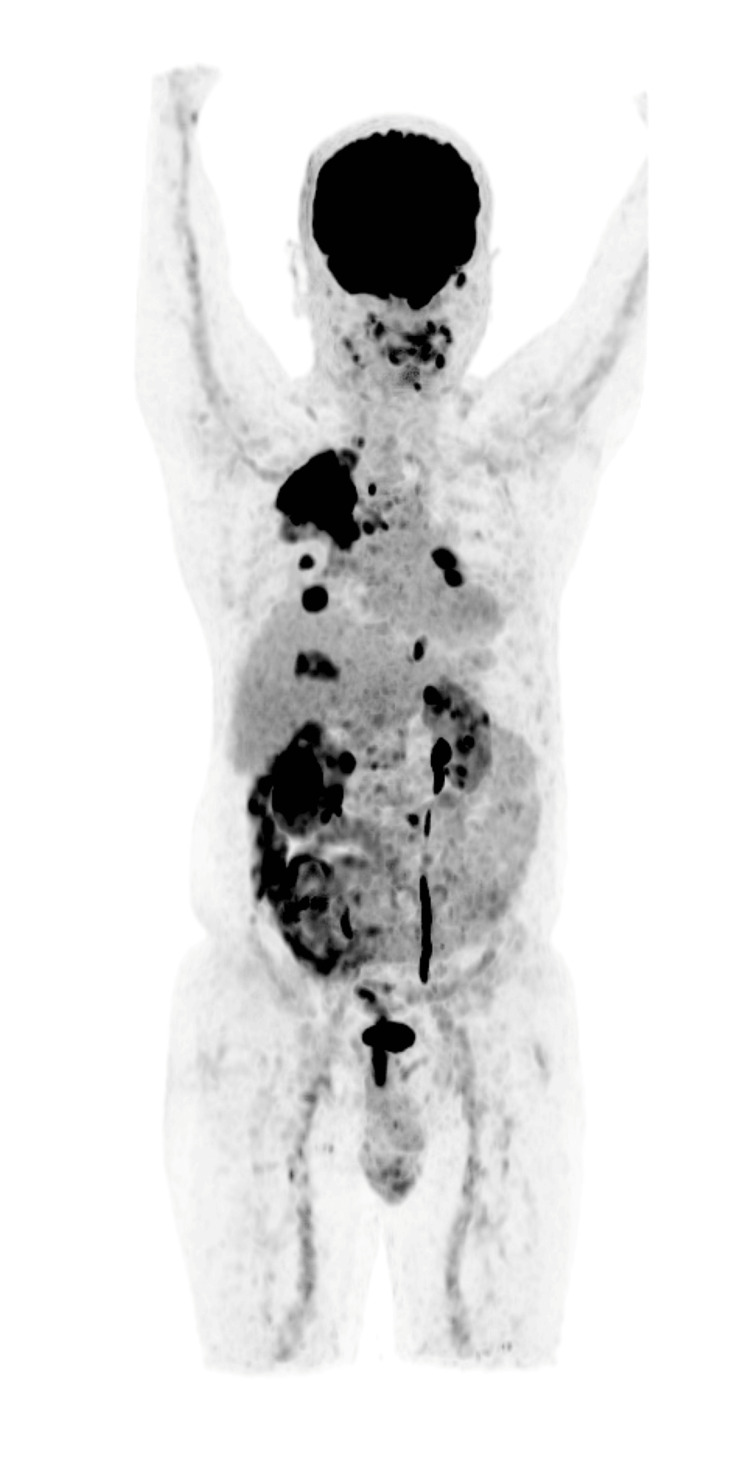
Maximum intensity projection(MIP) images showing all lesions together

**Figure 2:**
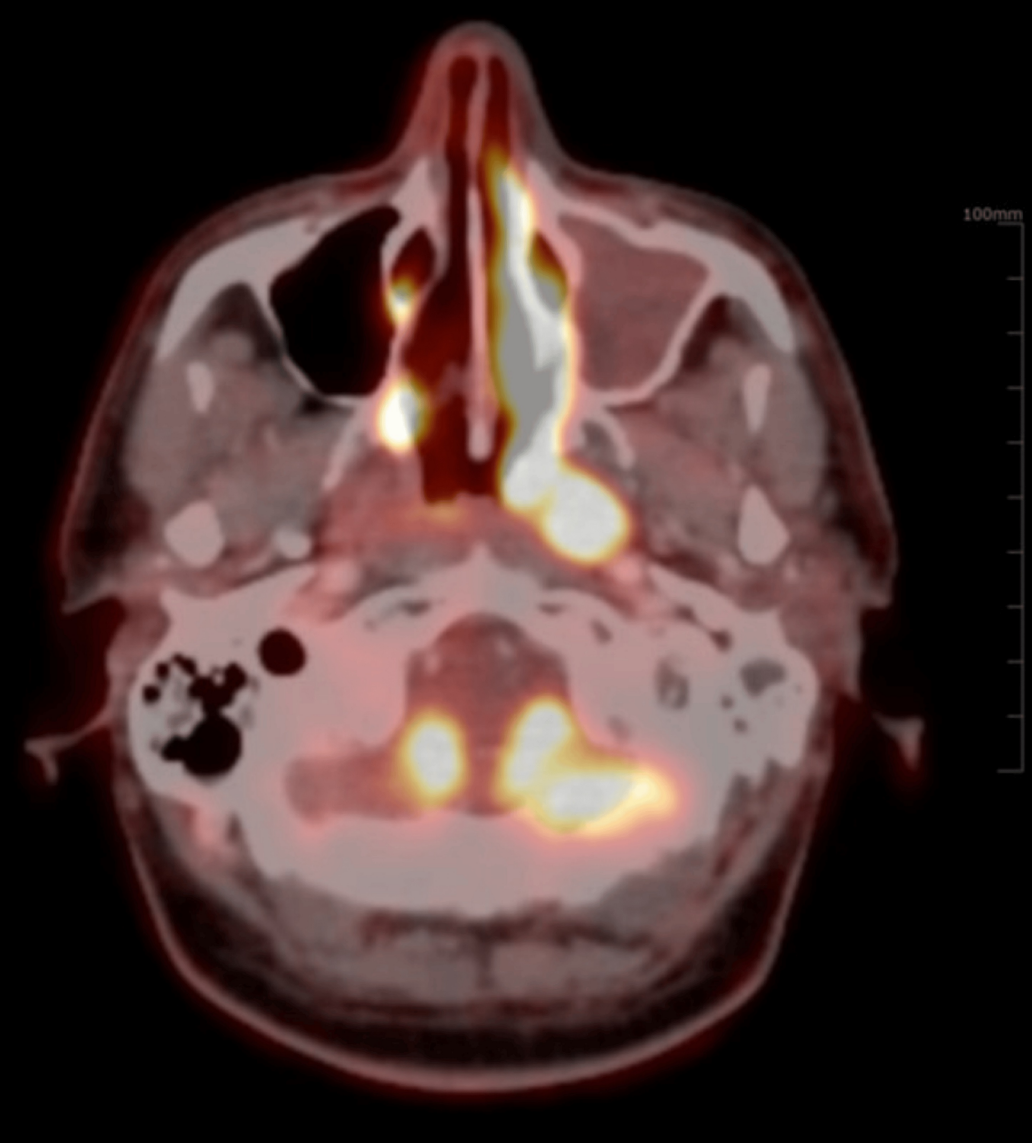
Transaxial image showing increased FDG concentration in the heterogeneously enhanced soft tissue mass in the left lateral wall of the nasopharynx, obliterating the Fossa of Rosenmuller and left Eustachian tube

**Figure 3:**
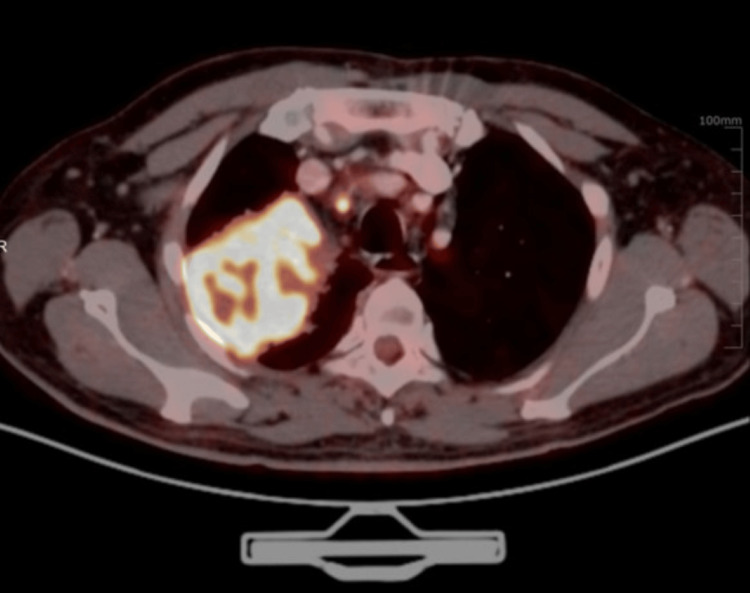
Transaxial images of the chest showing a FDG-concentrating irregular soft tissue mass lesion in the apical segment of the right lung upper lobe and the posterior costal pleura with FDG-concentrating satellite nodules, mediastinal, and bilateral hilar lymph nodes

**Figure 4:**
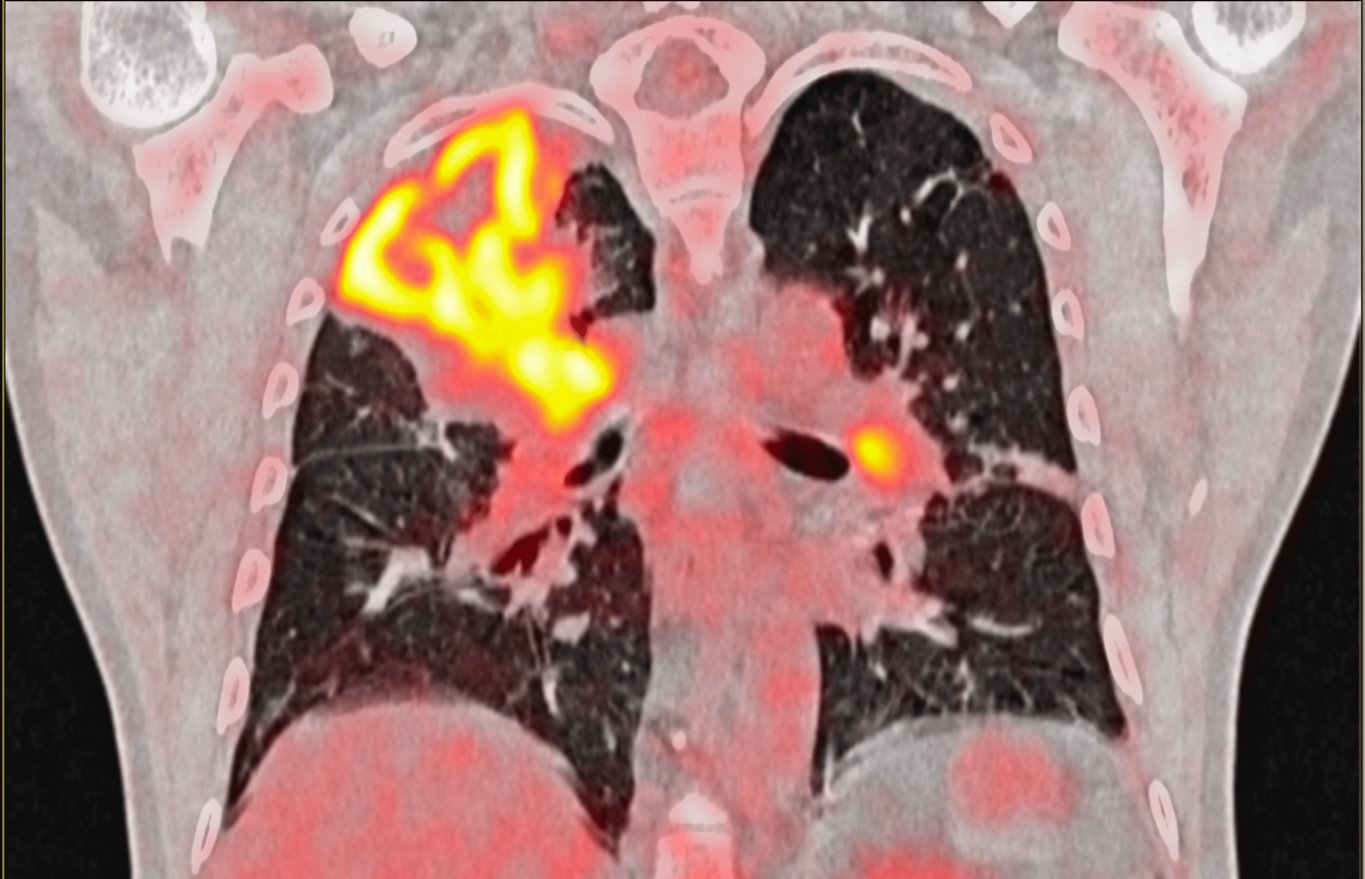
Coronal images of the chest show an FDG concentrating irregular soft tissue mass in the apical segment of the right lung upper lobe and the posterior costal pleura with FDG concentrating satellite nodules, mediastinal, and bilateral hilar lymph nodes

**Figure 5:**
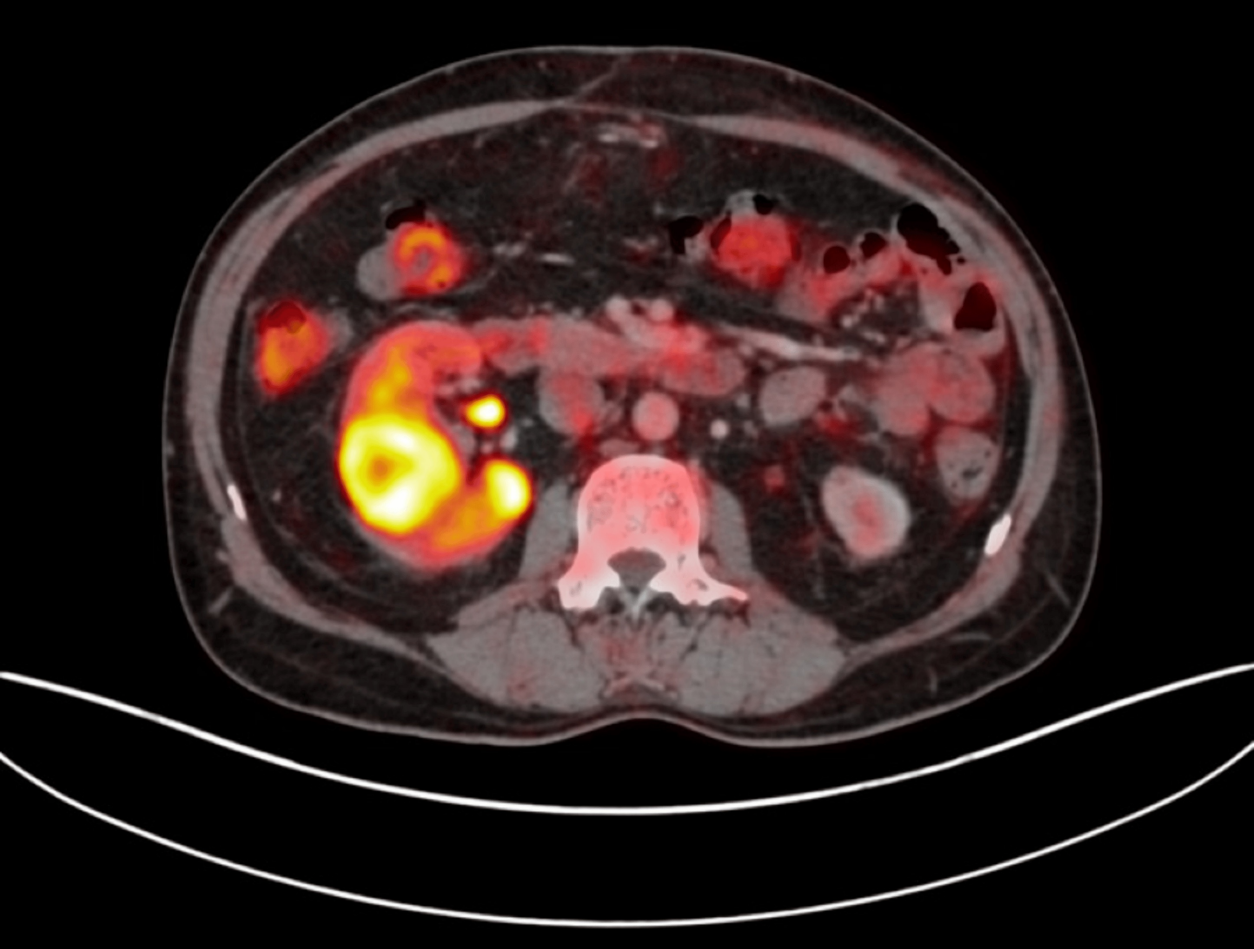
Transaxial images of the abdomen showing FDG concentration in heterogeneously hypoenhancing lesions in the right kidney

He presented here with dyspnea in addition to the previous symptoms he had. On examination, he appeared well with no evidence of respiratory distress. His vitals were within normal limits. On respiratory system examination, there were decreased breath sounds in the right infraclavicular area. Examination of other systems was unremarkable. He was advised to get a PET-CT done to assess the extent of the disease and to decide on a suitable site for tissue biopsy. PET-CT was done on 21st July 2023, which showed metabolically active, heterogeneously enhancing soft tissue mass present in the left lateral wall of the nasopharynx, obstructing the fossa of Rosenmuller, opening of the Eustachian tube and Torus tubarius measuring 19 mm × 13 mm, accompanied by metabolically active bilateral cervical, mediastinal, and right hilar lymph nodes. Additionally, there was a metabolically active soft tissue mass in the right upper lobe encasing the right upper lobe bronchus measuring 6.8 cm×7.5 cm×8 cm (anteroposterior × transverse × circumferential), along with multiple active nodules in both lungs and hypo-enhancing lesions involving both kidneys, suggesting metastasis (Figures 1-5).

The patient was investigated to rule out malignancy of the nasopharynx and bronchogenic carcinoma. He was taken up for fiber optic bronchoscopy (FOB) under conscious sedation. FOB showed hyperemic nodular growth occluding the right upper lobe bronchus, producing luminal narrowing, and also a similar lesion in the left upper lobe with partial luminal narrowing. EBUS (endobronchial ultrasound) showed enlarged nodes in stations 4R, 7, and 11L. Endobronchial biopsy from both lung lesions and EBUS FNAB (fine needle aspiration biopsy) was taken from station 7. A pediatric bronchoscope was passed intranasally in the same sitting, which showed soft tissue growth in the left lateral nasal wall, and a biopsy was taken.

**Figure 6:**
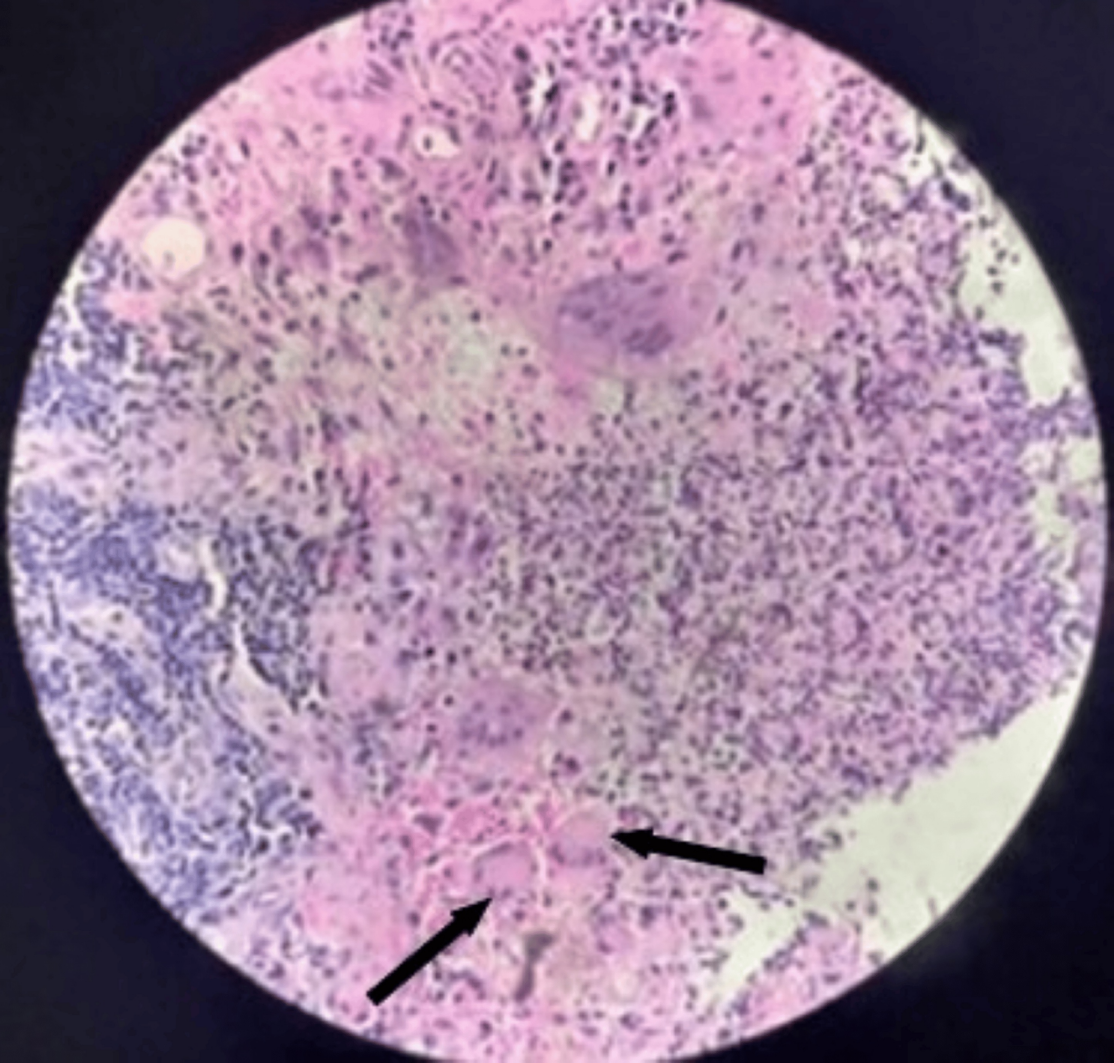
Endobronchial biopsy right upper lobe lesion showing granulomatosis with polyangiitis (hematoxylin and eosin stain). Histologic manifestations include necrotizing arteritis, small vessel vasculitis and capillaritis in the lung, and extravascular necrotizing granulomatous lesions. This photomicrograph shows granulomatous inflammation with giant cells (arrow) and surrounding acute and chronic inflammation

**Figure 7:**
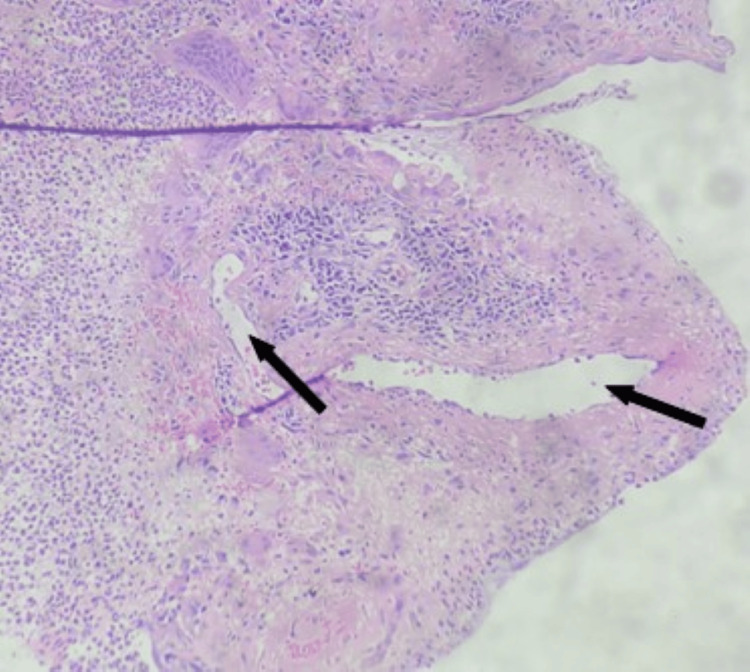
Endobronchial biopsy right upper lobe lesion (hematoxylin and eosin stain). This photomicrograph demonstrates necrotizing arteritis with fibrinoid necrosis (arrow) with surrounding giant cells and acute and chronic inflammation

Histopathology from both upper lobe lesions and nasopharyngeal growth showed the presence of granuloma with dense inflammatory infiltrate composed of neutrophils, lymphocytes, plasma cells, and eosinophils. Inflammation and fibrinoid necrosis of the vessel wall were also noted in these lesions. Histopathology from station 7 lymph node did not show granuloma or necrotic material. There was moderate cellularity and lymphocytes. A histopathological diagnosis of necrotizing granulomatous vasculitis was made (Figures 6, 7).

The differential diagnosis thought at this point was GPA, tuberculosis, and fungal infection. A Gene Xpert from the upper lobe lesion biopsy sample was negative for Mycobacterium tuberculosis.

A diagnostic nasal endoscopy was done. Tissue debris taken from the maxillary antrum showed groups of acute and chronic inflammatory cells. A nasal swab culture was taken to rule out fungal infection, but there was no growth. Discharge from ears was sent for culture, which showed no growth. Vasculitis serology revealed positive cytoplasmic antineutrophil cytoplasmic antibodies (c-ANCA) and increased proteinase 3 (PR3) antibodies (27.0 RU/ml, positive≥20) using serum enzyme immunoassay (EIA). A diagnosis of GPA was made. His BVAS (Birmingham vasculitis activity score) was 20/63 at this point. He was started on prednisolone 60 mg once daily with Rituximab 1 gram induction therapy on 30^th^ July 2023. He developed a right foot drop the next day. Intravenous methylprednisolone 500 mg once daily for three days was given in view of possible mononeuritis. Nerve conduction studies were done, which showed right peroneal nerve palsy, which improved after pulse methylprednisolone therapy. The second dose of Rituximab 1-gram infusion was given two weeks later, on 15 August 2023. Oral prednisolone was gradually tapered and stopped. A repeat chest X-ray on 16^th^ August 2023 showed a clearing of the right upper zone lesion. Meningococcal, influenza and pneumococcal vaccinations were advised. He was given antibiotic prophylaxis (in view of post-splenectomy status) along with pneumocystis jirovecii prophylaxis. An audiogram done on August 17, 2023, reported mild mixed hearing loss in the right ear and moderately severe mixed hearing loss in the left ear. The patient’s disease activity was reduced, and BVAS was 6/63 after treatment.

The patient was given a maintenance dose of Inj. Rituximab 500 mg six months later, on February 15, 2024. At this juncture, the patient was asymptomatic other than complaints of hearing loss. At the beginning of June 2024, he developed a left-sided headache with arthralgias and minimal bleeding from both ears. In view of the possibility of disease relapse, he was evaluated. His CT scan of paranasal sinuses showed mild to moderate pansinusitis with mild effacement of the right maxillary osteomeatal unit. The nasopharyngeal mass that was previously present had now disappeared. Patient PR3-ANCA was positive-28 RU/ml (positive≥ 20); the method used was serum EIA. He was diagnosed with a relapse of GPA. His BVAS was 9/63. He was induced with Rituximab 1 gram infusion in two doses on Day 1 and Day 15, respectively, as he opted for the same previous treatment regimen. He was also started on Mycophenolate 500 mg tablet twice daily. His symptoms, including headaches, arthralgias, and bleeding from the ears, have now resolved. His BVAS is 6/63 on 3^rd^ August 2023.

## Discussion

Granulomatosis with polyangiitis (GPA) is a form of small vessel vasculitis that typically presents with a triad of symptoms affecting the lungs, upper airways, and kidneys. Pulmonary involvement, which occurs in approximately 90% of cases, can range from mild, asymptomatic conditions to severe complications like diffuse alveolar hemorrhage [8].

The differential diagnosis for GPA is presented in Table 1 [9]. The most common causes of necrotizing granulomatous inflammation are infection, granulomatosis with polyangiitis (GPA), and aspiration pneumonia. Vascular inflammation (vasculitis) can occur in infectious granulomas, usually involving chronic inflammatory cells with or without granulomas [10]. However, true necrotizing vasculitis is characterized by fibrinoid necrosis of the media and is associated with necrotic neutrophils, which are not seen in infectious granulomas. This feature is specific to true vasculitis, such as GPA. Distinguishing features between infectious necrotizing granulomas and GPA include the presence of non-necrotizing granulomas and lymph node involvement by granulomatous inflammation in infections, which are typically not seen in GPA [10]. This case of granulomatosis with polyangiitis (GPA, Wegener's granulomatosis) can easily mimic a metastatic malignancy and has been rarely reported.

**Table 1:** Differential diagnosis

Differential diagnosis of Necrotizing Granulomatous Inflammation	
Infectious	Coccidioides immitis/Coccidioides posadasii, Cryptococcus neoformans/Cryptococcus gattii, Histoplasma capsulatum, Blastomyces dermatitidis, Aspergillus spp., Mucorales, Mycobacterium tuberculosis, non-tuberculous mycobacteria, Brucella spp., Nocardia spp., Yersinia spp., Bartonella henselae, Pneumocystis jiroveci, Echinococcus granulosus, Xanthogranulomatous pyelonephritis
Autoimmune causes	Granulomatosis with polyangiitis, Rheumatoid nodule, granuloma annulare, necrobiosis lipoidica
Other causes	Necrotizing sarcoid granulomatosis (NSG), infarct and lymphomatoid granulomatosis

Despite adequate treatment, AAV carries a significant risk of mortality and morbidity. The time between initial symptoms and actual diagnosis is positively correlated with disease outcome [11]. In the current case, the patient presented with various manifestations suggestive of nasopharynx and lung malignancy with metastasis. A biopsy and histopathological examination were indicative of necrotizing granulomatous vasculitis and suggested a GPA. A spiculated lung lesion invading the fissure, pleura, or diaphragm is mostly present in malignancy, but it can also be seen in GPA, which was similar to our study findings [6]. The most common radiographic presentation of GPA is of pulmonary masses and nodules, which are often multiple and cavitating, although a range of other pulmonary and pleural manifestations may occur [12]. In another study, a patient presented with an ANCA-positive GPA and subsequently developed lung cancer, which simulated a GPA relapse. ANCA serology was negative at the time of cancer diagnosis, suggesting immunological remission of vasculitis [13]. GPA is associated with an increased risk of malignancy, including the risk of pulmonary malignancy [14]. There are other studies that showed the initial diagnosis of metastatic cancer was later revised to granulomatosis with polyangiitis (GPA). The diagnosis was primarily established through a breast biopsy, which revealed characteristic findings of GPA. Following this accurate diagnosis, the patient received targeted treatment for GPA, leading to a full recovery [15-18].

In our case, our clinical suspicion of malignancy remained sufficiently high until serum PR3-ANCA came positive suggestive of GPA, and histopathological examination was suggestive of necrotizing granulomatous vasculitis [15,19]. Importantly, our patient satisfied the 2022 American College of Rheumatology/European Alliance of Associations for Rheumatology classification criteria for granulomatosis with polyangiitis, as detailed in Table 2. A score of ≥5 is needed for the classification of GPA. Our patient scored 11 [20].

**Table 2:** 2022 American College of Rheumatology/European Alliance of Associations for Rheumatology classification criteria for granulomatosis with polyangiitis Considerations when applying these criteria: These classification criteria should be applied to classify a patient as having granulomatosis with polyangiitis when a diagnosis of small or medium vessel vasculitis has been made. Alternate diagnosis mimicking vasculitis should be excluded before applying the criteria. Sum the scores for 10 items, if present. A score of ≥ 5 is needed for the classification of granulomatosis with polyangiitis.

Category	Criteria	Score
Clinical Criteria	Nasal involvement: bloody discharge, ulcers, crusting, congestion, blockage, or septal defect/perforation	+3
	Cartilaginous involvement: inflammation of ear or nose cartilage, hoarse voice or stridor, endobronchial involvement, or saddle nose deformity	+2
	Conductive or sensorineural hearing loss	+1
Laboratory, Imaging, and Biopsy Criteria	Positive test for cytoplasmic antineutrophil cytoplasmic antibodies (c-ANCA) or anti-proteinase 3 (anti-PR3) antibodies	+5
	Pulmonary nodules, mass, or cavitation on chest imaging	+2
	Granuloma, extravascular granulomatous inflammation, or giant cells on biopsy	+2
	Inflammation, consolidation, or effusion of the nasal/paranasal sinuses, or mastoiditis on imaging	+1
	Pauci-immune glomerulonephritis on biopsy	+1
	Positive test for perinuclear antineutrophil cytoplasmic antibodies (p-ANCA) or anti-myeloperoxidase (anti-MPO) antibodies	-1
	Blood eosinophil count ≥ 1 x 10⁹/L	-4

Learning points/take-home messages

It is important to consider a wide range of differentials, including GPA, in patients presenting with multiple masses, where the clinical picture is usually suggestive of a metastatic disease.

A tissue biopsy from an appropriate site with histopathological examination and positive ANCA serology is necessary to establish a diagnosis of GPA in unusual case scenarios.

Early diagnosis and treatment can alter the course of disease and help improve the prognosis of patients by preventing damage to one or more organs.

## Conclusions

This case illustrates the critical importance of differentiating GPA from metastatic malignancy, which is essential for selecting appropriate treatment and improving patient outcomes. Despite presenting symptoms and imaging findings suggestive of metastatic cancer, the definitive diagnosis of GPA was confirmed through biopsy and serological testing for ANCA antibodies. The patient responded well to the combined immunosuppressive therapy of high-dose prednisolone and rituximab, achieving low disease activity. This case highlights the necessity for a comprehensive diagnostic approach, including histopathological examination and ANCA serology, to avoid misdiagnosis and to initiate timely and effective treatment. Early and accurate diagnosis of GPA can prevent unnecessary interventions and significantly alter the disease course, underscoring the importance of considering GPA in the differential diagnosis of pulmonary and nasopharyngeal masses.
